# Морфологические предикторы эффективности терапии митотаном при адренокортикальном раке

**DOI:** 10.14341/probl13172

**Published:** 2022-12-20

**Authors:** А. В. Ткачук, Д. Г. Бельцевич, Э. Э. Порубаева, Л. С. Урусова

**Affiliations:** ФГБУ "Национальный медицинский исследовательский центр эндокринологии" Минздрава России; ФГБУ "Национальный медицинский исследовательский центр эндокринологии" Минздрава России; ФГБУ "Национальный медицинский исследовательский центр эндокринологии" Минздрава России; ФГАОУ ВО «Первый Московский государственный медицинский университет им. И.М. Сеченова» Минздрава России (Сеченовский университет); ФГБУ "Национальный медицинский исследовательский центр эндокринологии" Минздрава России

**Keywords:** адренокортикальный рак, АКР, митотан, предикторы, эффективность терапии, безрецидивная выживаемость, SOAT1, CYP2W1, RRM1

## Abstract

ОБОСНОВАНИЕ. Адренокортикальный рак (АКР) является орфанной злокачественной опухолью коры надпочечника с преимущественно неблагоприятным прогнозом и агрессивным клиническим течением. На сегодняшний день митотан представляет собой безальтернативный по эффективности лекарственный препарат в терапии АКР. Поиск прогностических параметров, определяющих чувствительность АКР к проводимому лечению, в настоящее время является актуальной задачей. В качестве потенциальных предикторов ответа на терапию митотаном рассматриваются уровни экспрессии большой субъединицы рибонуклеотидредуктазы М1 (RRM1), цитохрома P450 2W1 (CYP2W1) и стерол-O-ацилтрансферазы-1 (SOAT1).ЦЕЛЬ. Оценить иммуногистохимическую экспрессию RRM1, CYP2W1 и SOAT1 в АКР в качестве маркеров клинического исхода и ответа на терапию митотаном.МАТЕРИАЛЫ И МЕТОДЫ. В исследование были включены 62 пациента старше 17 лет с диагнозом АКР, подтвержденным гистологически и иммуногистохимически. 29 пациентам в послеоперационном периоде была инициирована терапия митотаном, 33 пациента находились на динамическом наблюдении без сопутствующего лекарственного лечения. Для иммуногистохимического исследования использовались антитела к RRM1, CYP2W1, SOAT1 с разведением в соответствии с рекомендациями фирмы-производителя.РЕЗУЛЬТАТЫ. В группе пациентов с низкой и умеренной иммунореактивностью RRM1, CYP2W1 и SOAT1 в опухоли и отсутствием противоопухолевого лечения отмечена лучшая безрецидивная выживаемость (БРВ) (p=0,037, p=0,020 и p=0,001 соответственно) в сравнении с группой пациентов, получающих терапию митотаном при данном уровне экспрессии маркеров. При высокой иммунореактивности маркеров статистически значимые различия в БРВ не обнаружены.ЗАКЛЮЧЕНИЕ. В соответствии с результатами нашего исследования низкая экспрессия RRM1, CYP2W1 и SOAT1 ассоциировалась с худшей БРВ при противоопухолевом лечении. Результаты работы свидетельствуют о необходимости оценки уровней иммунореактивности данных маркеров у пациентов с АКР перед началом лечения митотаном с целью прогнозирования эффективности терапии.

## Abstract (en)

BACKGROUND: Adrenocortical cancer (ACC) is an orphan malignant tumor of the adrenal cortex with a predominantly poor prognosis and an aggressive clinical course. Nowadays, mitotane is a non-alternative drug in the treatment of ACC. The search for prognostic parameters that determine the sensitivity of ACC to ongoing treatment is currently an urgent task. Expression levels of the large subunit of ribonucleotide reductase M1 (RRM1), cytochrome P450 2W1 (CYP2W1), and sterol- O-acyltransferase-1 (SOAT1) are considered as potential predictors of response to mitotane therapy.AIM: To assess the immunohistochemical expression of RRM1, CYP2W1 and SOAT1 in ACC as markers of clinical outcomes and response to the therapy with mitotane.MATERIALS AND METHODS: The study included 62 patients older than 17 years of age with a diagnosis of ACC confirmed histologically and immunohistochemically. Mitotane therapy was initiated in 29 patients in the postoperative period, 33 patients were under dynamic observation without concomitant drug treatment. Antibodies to RRM1, CYP2W1, SOAT1 were used diluted in accordance with recommendations of firms-manufacturers for immunohistochemical detection. RESULTS: In the group of patients with low and moderate RRM1, CYP2W1 and SOAT1 immunoreactivity in the tumor and no antitumor therapy, a better DFS was noted (p=0.037, p=0.020 and p=0.001, respectively) compared to the group of patients receiving mitotane therapy at this level of marker expression. With high immunoreactivity of the markers, no statistically significant differences in DFS were found.CONCLUSION: Consistent with the findings in our study, low expression of RRM1, CYP2W1 and SOAT1 was associated with worse DFS with antitumor therapy. The results of the work indicate the need to assess the levels of immunoreactivity of these markers in patients with ACC before starting treatment with mitotane in order to predict the efficiency of therapy.

## ОБОСНОВАНИЕ

Адренокортикальный рак (АКР) является редким злокачественным новообразованием коры надпочечника с распространенностью от 0,7 до 2 случаев на 1 млн населения в год [1][2]. Несмотря на то что в структуре смертности от онкологических новообразований на АКР приходится только 0,04–0,2%, данное заболевание является вторым после анапластической карциномы щитовидной железы наиболее агрессивным злокачественным новообразованием эндокринной системы. Так, по классификации Европейской рабочей группы по изучению опухолей надпочечников (ENSAT), пятилетняя общая выживаемость (ОВ) при поздних стадиях заболевания составляет менее 30% [1][3][4]. Вместе с тем АКР является гетерогенным заболеванием с трудно предсказуемым исходом: встречаются как клинически индолентные новообразования, так и крайне агрессивные опухоли с летальным исходом в течение менее одного года. На момент постановки диагноза у большинства пациентов наблюдается как локорегионарное, так и отдаленное метастазирование опухоли. В настоящее время единственным возможным вариантом излечения при АКР является его радикальное хирургическое удаление, в то время как на поздних стадиях заболевания выполнение оперативного пособия часто невозможно. Оперированным пациентам в объеме R0 рекомендуется адъювантная терапия митотаном; больным с неоперабельным, или метастатическим, или прогрессирующим после оперативного лечения заболеванием рекомендуется полихимиотерапия, одним из компонентов которой является митотан.

Митотан (1-(2-хлорфенил)-1-(4-хлорфенил)-2,2-дихлорэтан (o,p’-DDD)), производное дихлордифенилтрихлорэтана, является безальтернативным по эффективности лекарственным препаратом в терапии АКР, который одобрен EMA (European Medicine Agency) и FDA (Agency of Food and Drug Administration, USA), а с 2018 г. зарегистрирован и разрешен к применению в Российской Федерации.

Механизм действия митотана основан на избирательном повреждении ткани коры надпочечника, а именно на цитолитической активности в отношении его пучковой и сетчатой зон. Митотан оказывает токсическое воздействие на митохондрии опухолевых клеток за счет влияния на белковый комплекс IV, что приводит к фрагментации митохондриальных мембран и нарушению функционирования дыхательной цепи электронов [5]. Кроме того, в ряде недавних исследований продемонстрировано влияние митотана на фермент стерол-O-ацилтрансферазу-1 (SOAT1). Инактивация SOAT1 вызывает накопление свободного холестерина и жирных кислот и развитие индуцированного липидами стресса эндоплазматического ретикулума (ЭПР-стресс), который инициирует апоптоз опухолевых клеток [6]. Предполагается, что ингибирующее влияние митотана на стероидогенез обусловлено несколькими механизмами. Митотан вызывает снижение уровня мРНК цитохромов CYP11A1 и CYP17A1, кодирующих белки, которые участвуют в биосинтезе кортизола и дегидроэпиандростеронсульфата, ингибирует ферменты CYP21A2, CYP11B1, снижает экспрессию регуляторной субъединицы цАМФ-зависимой протеинкиназы (PRKAR1A) и гена трансформирующего фактора роста бета-1. Кроме того, каскад процессов, возникающий в результате ингибирования SOAT1, приводит к подавлению стероидогенного регуляторного гена — транскрипционного фактора 1, связывающего регуляторный элемент стерола (SREBF). В результате происходит активация каспазы 3, что инициирует запуск внутреннего пути апоптоза. Таким образом, ингибирование SREBF посредством ЭПР-стресса может лежать в основе одногоиз механизмов, с помощью которых митотан ингибирует стероидогенез [7–9].

Противоопухолевый эффект митотана напрямую коррелирует с его уровнем в плазме крови: достижение целевой концентрации, равной 14 мг/л, считается наиболее значимым предиктором ответа на терапию и ассоциируется с более длительным периодом безрецидивной выживаемости (БРВ). Узкий терапевтический диапазон митотана с необходимостью тщательного мониторинга его концентрации в плазме крови, крайне высокая токсичность с развитием широкого спектра побочных эффектов и ограниченная эффективность терапии обуславливают необходимость поиска прогностических параметров, определяющих чувствительность опухоли к проводимому лечению.

В качестве потенциальных предикторов ответа на терапию митотаном рассматриваются уровни экспрессии большой субъединицы рибонуклеотидредуктазы М1 (RRM1), цитохрома P450 2W1 (CYP2W1) и SOAT1. RRM1 представляет собой мультимерный фермент, участвующий в синтезе и репарации ДНК, регуляции пролиферации и миграции клеток. В работе 2012 г. М. Volante и соавт. продемонстрировали корреляцию экспрессии гена RRM1 с БРВ и ОВ у пациентов с АКР: низкая экспрессия гена RRM1 у пациентов, получавших митотан в адъювантом режиме, ассоциировалась с более длительным периодом БРВ по сравнению с группой пациентов, которые находились на динамическом контроле [10].

SOAT1 в различной степени обнаруживается в большинстве типов клеток и тканей организма, при этом наибольший уровень экспрессии фермента отмечен в клетках коры надпочечников, где он кодирует соответствующий белок и является мишенью стероидогенного фактора-1 (SF-1). SOAT1 участвует в обеспечении гомеостаза внутриклеточного холестерина и защите клеток надпочечников от потенциально опасного воздействия избытка свободного холестерина [6][11]. По последним данным, высокая экспрессия SOAT1 в злокачественных новообразованиях ассоциирована с низкой ОВ и неблагоприятным прогнозом, в том числе и при АКР [11]. Тем не менее в одной из работ была обнаружена взаимосвязь противоопухолевой терапии митотаном с более длительным периодом до прогрессирования заболевания в случае высокой экспрессии SOAT1 [6].

Установлено, что мРНК и/или белок CYP2W1 экспрессируются внутриутробно в процессе развития желудочно-кишечного тракта плода и инактивируются после рождения. Активация экспрессии, связанная с деметилированием, обнаруживается при колоректальном раке, гепатоцеллюлярной карциноме, раке легкого и груди, рабдомиосаркоме у детей, а также в случае АКР [12]. Выраженная иммунореактивность CYP2W1 в колоректальном раке наблюдается в трети случаев и свидетельствует о неблагоприятном прогнозе, в то время как при терапии митотаном в АКР она ассоциирована с более длительным периодом БРВ [13].

## ЦЕЛЬ ИССЛЕДОВАНИЯ

Оценить иммуногистохимическую экспрессию RRM1, CYP2W1 и SOAT1 в АКР в качестве маркеров клинического исхода и ответа на терапию митотаном.

## МАТЕРИАЛЫ И МЕТОДЫ

## Место и время проведения исследования

Место проведения. Всем пациентам была проведена адреналэктомия в НМИЦ эндокринологии Минздрава России (руководитель отдела хирургии — Кузнецов Н.С., заведующий отделом онкоэндокринологии — Бельцевич Д.Г.), а также в других лечебных учреждениях. Нами выполнялось патоморфологическое исследование операционного материала пациентов, получивших лечение в ФГБУ «НМИЦ эндокринологии» Минздрава России, а также повторное патоморфологическое исследование (пересмотр) консультативного материала из других лечебных учреждений на базе отдела фундаментальной патоморфологии ФГБУ «НМИЦ эндокринологии» Минздрава России. Всем пациентам, получающим терапию митотаном, проводилось регулярное мониторирование концентрации данного препарата в плазме крови. У всех пациентов концентрация митотана в крови достигала целевого уровня 14–20 мкг/л.

Время исследования. Исследование проводилось в период с 2005 по 2020 г.

## Изучаемые популяции (одна или несколько)

Основная группа — пациенты с АКР, которым в послеоперационном периоде была инициирована терапия митотаном.

Контрольная группа — пациенты, которые находились на динамическом наблюдении без сопутствующего лекарственного лечения.

Критерии включения в основную группу.

## Способ формирования выборки из изучаемой популяции (или нескольких выборок из нескольких изучаемых популяций)

Сплошной.

## Дизайн исследования

Ретроспективное одноцентровое наблюдательное исследование.

## Методы

Диагноз АКР подтвержден с помощью иммуногистохимического (ИГХ) исследования, каждый случай классифицирован в соответствии с 4-м изданием Классификации опухолей эндокринных органов (WHO Classification of Tumors Pathology and Genetics, 2017) на один из вариантов — классический, онкоцитарный, миксоидный (саркоматоидный вариант АКР диагностирован не был) [14]. Проведен анализ доступных клинических данных, включающих результаты гормонального и инструментального обследования.

ИГХ-исследование проводилось на срезах толщиной 3 мкм, расположенных на стеклах с полилизиновым слоем (Leica, Германия). Исследование осуществлялось на полностью автоматизированном иммуногистостейнере Leica Bond III (Германия), позволяющем депарафинизировать срезы, проводить инкубацию с антителами при постоянной заданной температуре, выполнять энзиматическую демаскировку антигенов, высокотемпературную демаскировку антигенов в буферах pH 6,0 и 8,8, подкрашивать препараты гематоксилином. Исследование проводилось по стандартным протоколам, рекомендованным фирмой-производителем.

Для ИГХ-исследования использовались антитела к RRM1 (поликлональные кроличьи антитела к RRM1 Thermo, 100 мкл, PA5-32574), CYP2W1 (поликлональные кроличьи антитела к CYP2W1 Thermo, 100 мкл, PA5-50389), SOAT1 (поликлональные кроличьи антитела к SOAT1, 100 мкл, Abcam, ab217923). Разведение антител осуществлялось в соответствии с рекомендациями фирмы-производителя. Экспрессия антител оценивалась в цитоплазме и ядрах опухолевых клеток по степени интенсивности: 1–20% — 1 балл; 20–70% — 2 балла; 70–100% — 3 балла.

## Статистический анализ

Для анализа связи БРВ с изучаемыми факторами (экспрессия RRM1, CYP2W1 и SOAT1 в АКР) выполнен независимый и мультивариантный регрессионный анализ с включением факторов в анализ методом Каплана–Мейера с последующими попарными сравнениями методом log-rank тест. Различия признаны статистически достоверными при p<0,05. Проверка гипотезы о различной БРВ в подгруппах по митотану при разном уровне экспрессии факторов с поправкой на стадию по ENSAT выполнена с помощью модели пропорциональных рисков (Cox regression). Для непосредственных статистических расчетов использовали программное обеспечение IBM SPSS Statistics версия 26.0.

## Этическая экспертиза

Учитывая ретроспективный дизайн научной работы, Комитет по биомедицинской этике постановил: при условии публикации данных в деперсонифицированном виде исследование в этической экспертизе не нуждается (номер протокола 10, дата подписания 26.05.2020).

## РЕЗУЛЬТАТЫ

В исследование были включены 62 пациента с АКР: 40 (64,5%) женщин и 22 (36,5%) мужчины в возрасте от 17 до 82 лет, с медианой возраста для мужчин 36,5 года и 51,5 года для женщин. В послеоперационном периоде 29 пациентам была инициирована терапия митотаном ввиду принадлежности больных к группе высокого риска рецидива/прогрессирования АКР (индекс пролиферативной активности Ki-67 по результатам ИГХ-исследования более 10%), 33 пациента были отнесены кгруппе с низким/промежуточным риском рецидива/прогрессирования АКР и находились на динамическом наблюдении без сопутствующего лекарственного лечения. Клинические и морфологические характеристики пациентов представлены в таблице 1.

**Table:** Таблица 1. Клинические и морфологические характеристики пациентов с адренокортикальным раком

	Терапия митотаномN=29	Динамическое наблюдениеN=33
Клинические характеристики
Возраст (медиана (мин-макс)), годы	45 (17–74)	48 (21–82)
Размер образования (медиана (мин-макс)), см	8,5 (3,5–25,0)	8,0 (4,0–25,0)
Стадия, n (%)		
I	3 (10,3)	6 (18,2)
II	10 (34,5)	18 (54,5)
III	13 (44,8)	9 (27,3)
IV	3 (10,3)	0 (0)
Функциональная активность, n (%)		
Гиперкортицизм	11 (37,9)	8 (24,2)
Гиперандрогения	0 (0)	0 (0)
Гиперальдостеронизм	1 (3,4)	0 (0)
Смешанная	2 (6,9)	1 (3,0)
Период наблюдения (медиана (мин-макс)), мес	32 (5–190)	37 (4–159)
Гистопатологические характеристики
Weiss (медиана (мин-макс))	6 (5–9)	6 (4–9)
Параметры шкалы Weiss, n (%)		
Инвазия в вены	7 (24,1)	5 (15,2)
Инвазия в синусоиды	12 (41,4)	14 (42,4)
Инвазия в капсулу	19 (65,5)	19 (57,6)
Патологические митозы	27 (93,1)	26 (78,8)
Участки некроза	26 (89,7)	23 (69,7)
Высокий ядерный индекс	16 (55,2)	20 (60,6)
Менее 25% клеток со светлой цитоплазмой	27 (93,1)	29 (87,9)
Диффузный рост	24 (82,8)	24 (72,7)
Более 5 митозов на 50 полей зрения при большом увеличении	0 (0)	3 (9,1)
Иммуногистохимические характеристики
Индекс Ki-67% (медиана (мин-макс))	25 (7–60)	10 (5–40)
Морфологический вариант, n (%)
Классический	24 (82,8)	20 (60,6)
Миксоидный	3 (10,3)	4 (12,1)
Онкоцитарный	2 (6,9)	9 (27,3)
Ответ на лечение (n (%) отсутствия рецидивов за время наблюдения)
	8 (27,6)	18 (54,5)

Проанализирована экспрессия потенциальных прогностических параметров, определяющих чувствительность опухоли к терапии митотаном, в зависимости от морфологических вариантов АКР (рис. 1, 2).

**Figure:**
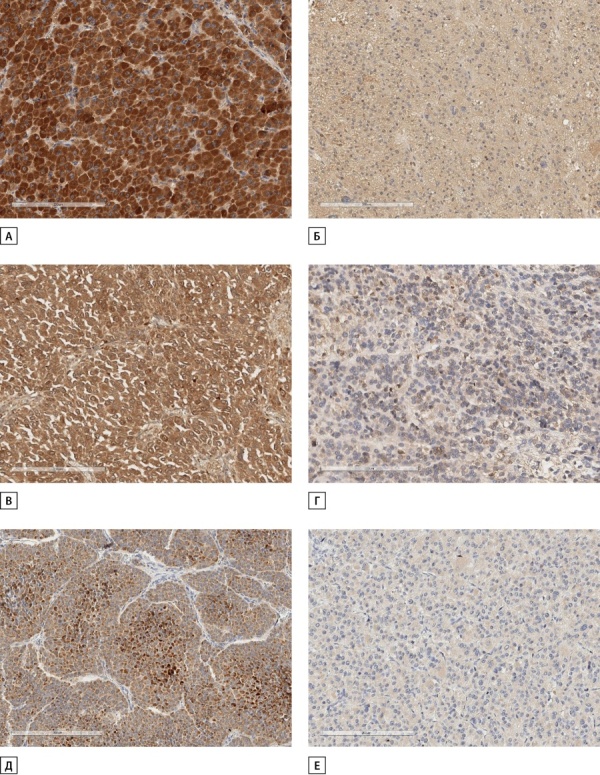
Рисунок 1. Иммунореактивность SOAT1, CYP2W1 и RRM1 в вариантах адренокортикального рака. А. Выраженная иммуногистохимическая экспрессия SOAT1 в онкоцитарном варианте АКР.Б. Слабая иммуногистохимическая экспрессия SOAT1 в онкоцитарном варианте АКР.В. Выраженная иммуногистохимическая экспрессия CYP2W1 в миксоидном варианте АКР.Г. Слабая иммуногистохимическая экспрессия CYP2W1 в классическом варианте АКР.Д. Выраженная иммуногистохимическая экспрессия RRM1 в классическом варианте АКР.Е. Слабая иммуногистохимическая экспрессия RRM1 в классическом варианте АКР.

**Figure:**
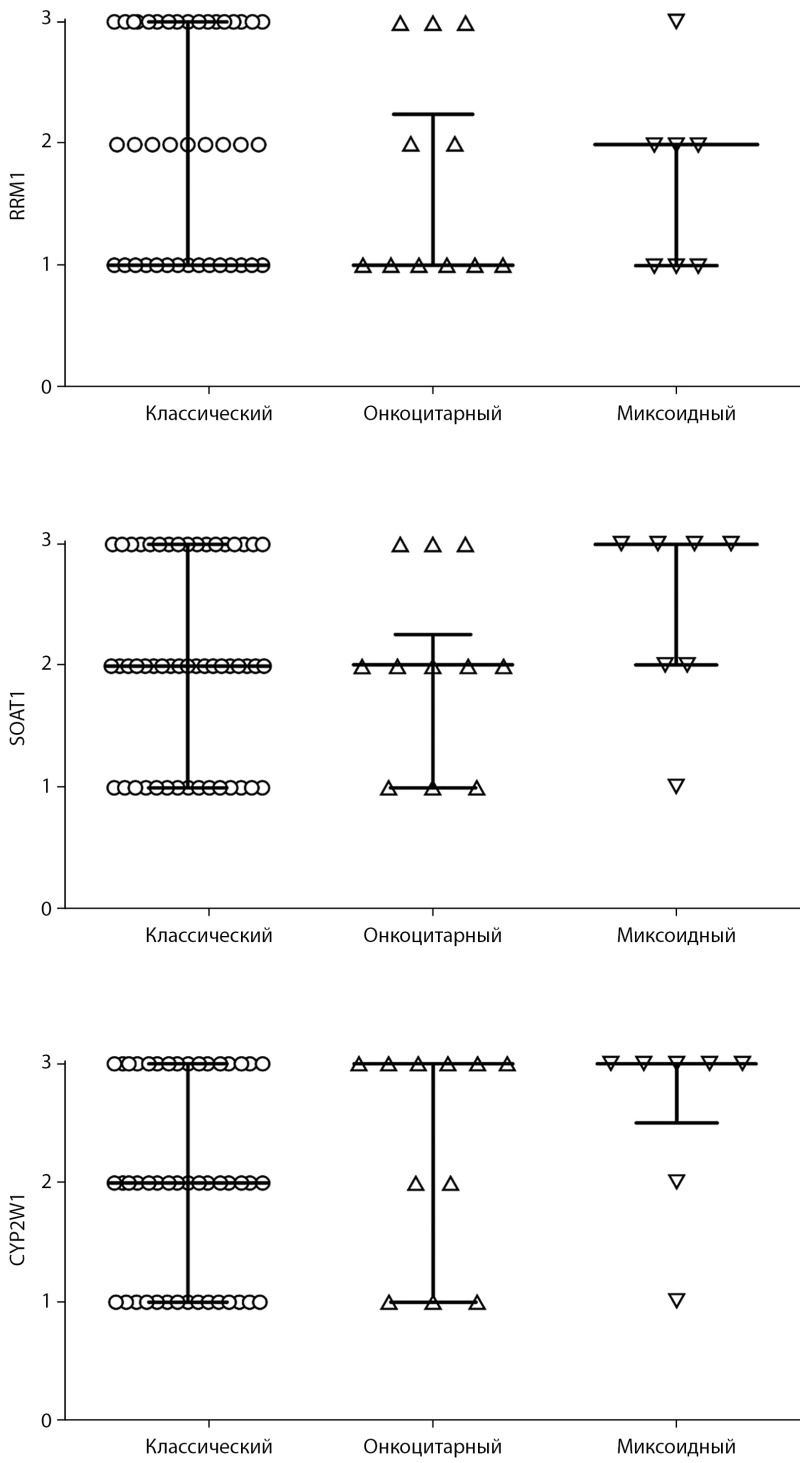
Рисунок 2. Распределение иммунореактивности SOAT1, CYP2W1 и RRM1 в вариантах адренокортикального рака.

В классическом варианте АКР выявлено относительно равномерное распределение уровней иммунореактивности SOAT1, CYP2W1 и RRM1. Экспрессия SOAT1 в онкоцитарном варианте характеризовалась преимущественно умеренной и низкой иммунореактивностью, в то время как в уровнях экспрессии CYP2W1 и RRM1 отмечено обратно пропорциональное распределение. Миксоидный вариант отличался выраженной иммунореактивностью SOAT1 и CYP2W1, при этом отмечена преимущественно низкая и умеренная экспрессия RRM1.

Первоначальный анализ БРВ у пациентов с АКР, получающих терапию митотаном и находящихся на динамическом наблюдении, произведен без учета уровней экспрессии вышеописанных маркеров (рис. 3).

**Figure:**
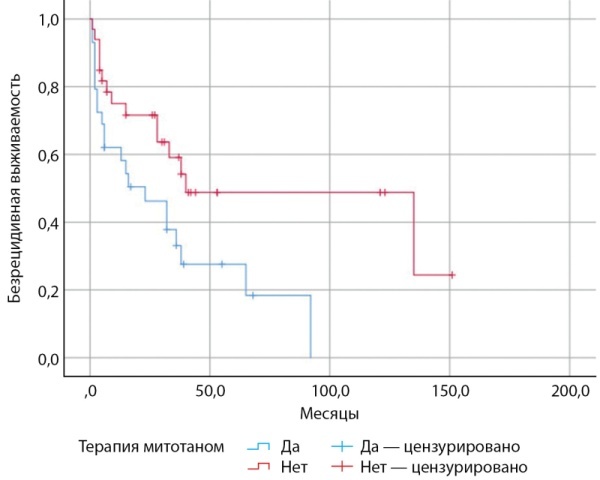
Рисунок 3. Безрецидивная выживаемость у пациентов с адренокортикальным раком, получающих терапию митотаном и находящихся на динамическом наблюдении, без учета уровней экспрессии SOAT1, CYP2W1 и RRM1.

При включении в анализ уровней экспрессии потенциальных прогностических маркеров обнаружено, что низкая экспрессия RRM1 в опухоли и отсутствие терапии митотаном в анамнезе ассоциировались с лучшей БРВ (p=0,037) (рис. 4, 5) в сравнении с пациентами, получающими терапию митотаном, при данном уровне экспрессии маркера. В случаях умеренной и высокой экспрессии RRM1 статистически значимых различий БРВ в обеих группах пациентов выявлено не было.

**Figure:**
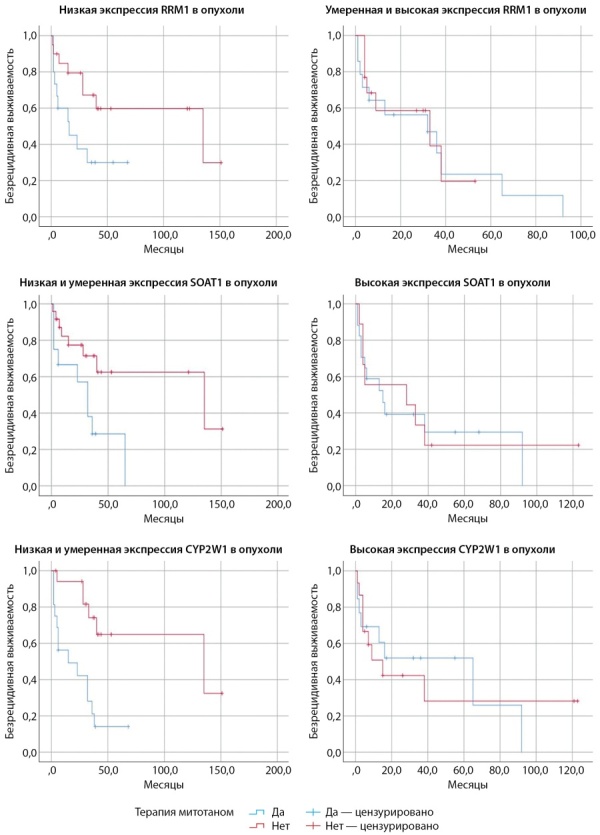
Рисунок 4. Безрецидивная выживаемость у пациентов с адренокортикальным раком, получающих терапию митотаном и находящихся на динамическом наблюдении, в зависимости от экспрессии RRM1, SOAT1, CYP2W1.

**Figure:**
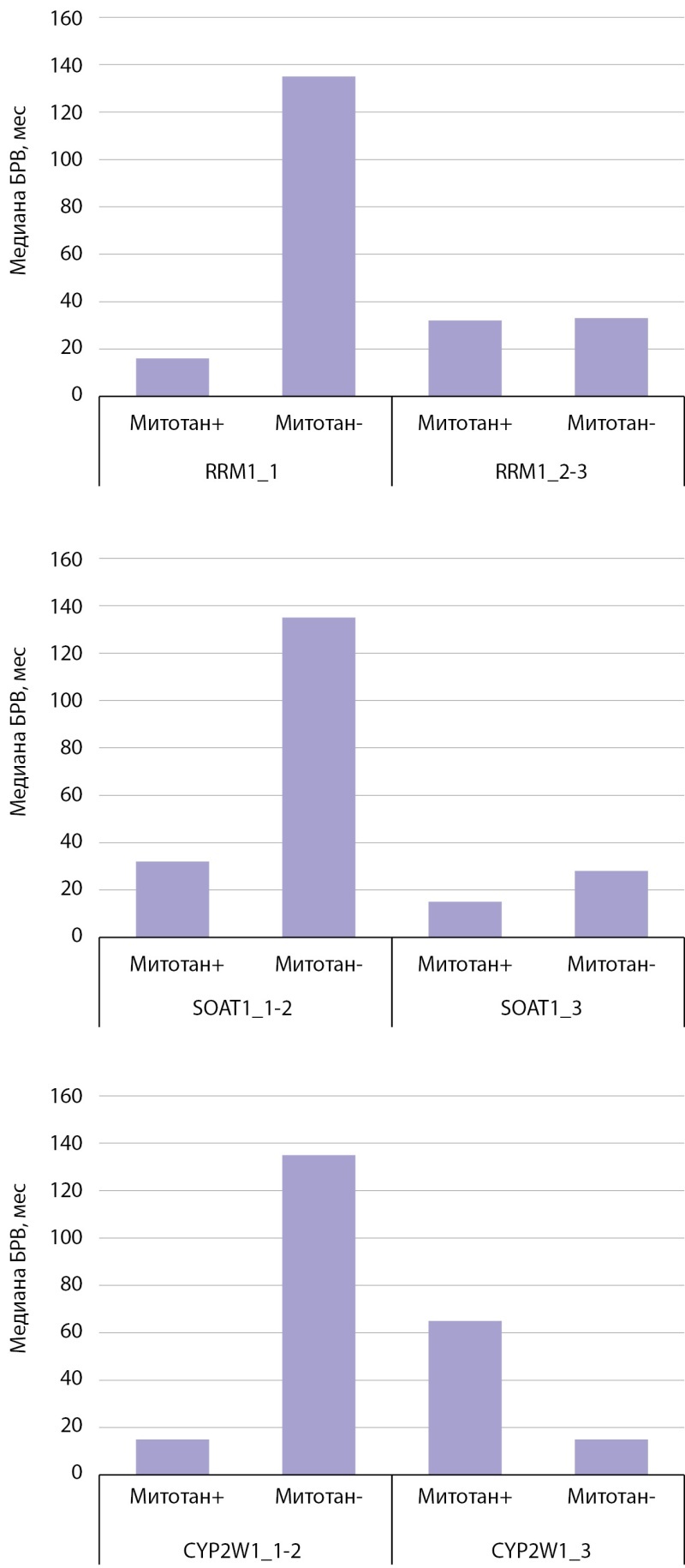
Рисунок 5. Столбчатые диаграммы, отображающие безрецидивную выживаемость у пациентов с адренокортикальным раком, получающих терапию митотаном и находящихся на динамическом наблюдении, в зависимости от экспрессии RRM1, SOAT1, CYP2W1.

В случае низкой и умеренной экспрессии SOAT1 БРВ у пациентов с АКР, не получающих лечение, была статистически достоверно благоприятнее (p=0,020) (рис. 4, 5), чем у пациентов с терапией. При высокой экспрессии маркера статистически значимые различия не обнаружены.

Результаты анализа CYP2W1 аналогичны: у пациентов с низкой и умеренной иммунореактивностью, которые не получали митотан, наблюдалась лучшая БРВ (p=0,001) (рис. 4, 5) по сравнению с пациентами, находящимися на лечении. Статистически значимых различий БРВ в обеих группах пациентов при высокой экспрессии CYP2W1 не выявлено.

Далее для того, чтобы устранить влияние стадии ENSAT на БРВ пациентов, была выполнена двухфакторная Cox-регрессия со стратификацией по уровню экспрессии обсуждаемых ИГХ-маркеров. Результаты регрессионного анализа с поправкой на стадию ENSAT представлены в таблицах 2–4.

При анализе иммунореактивности RRM1, SOAT1, CYP2W1 с учетом поправки на стадию ENSAT обнаружено, что при низком и умеренном уровне экспрессии CYP2W1 у пациентов, получающих терапию митотаном, риск рецидива был в 4,28 раза выше (р=0,011), чем у пациентов без лечения (табл. 2). Аналогичная тенденция в повышении риска рецидива более чем в 2 раза выявлена при низкой и умеренной экспрессии SOAT1 (р=0,062) (табл. 3) и RRM1 (p=0,10) (табл. 4); однако учитывая то, что статическая значимость не достигается, можно говорить только о вероятностном характере события. Во всех трех группах с высокой экспрессией обсуждаемых маркеров при внесении поправки на стадию ENSAT обнаруживается, что митотан оказывает благоприятный эффект на БРВ (значение ExpB менее 1). Вместе с тем статическая значимость также не достигается, поэтому можно отметить только наличие тенденции.

**Table:** Таблица 2. Влияние уровня экспрессии CYP2W1 на безрецидивную выживаемость у пациентов, получающих митотан, с поправкой на стадию ENSAT

Уровень экспрессии CYP2W1	W	Exp (B)	р
Низкий и умеренный	Терапия митотаном	6,48	4,28	0,01
Стадия ENSAT	1,55	0,53	0,21
Высокий	Терапия митотаном	0,01	0,97	0,95
Стадия ENSAT	0,06	0,88	0,80

**Table:** Таблица 3. Влияние уровня экспрессии SOAT1 на безрецидивную выживаемость у пациентов, получающих митотан, с поправкой на стадию ENSAT

Уровень экспрессии SOAT1	W	Exp (B)	р
Низкий и умеренный	Терапия митотаном	3,48	2,66	0,06
Стадия ENSAT	1,24	0,56	0,27
Высокий	Терапия митотаном	0,09	0,84	0,77
Стадия ENSAT	0,77	0,60	0,38

**Table:** Таблица 4. Влияние уровня экспрессии RRM1 на безрецидивную выживаемость у пациентов, получающих митотан, с поправкой на стадию ENSAT

Уровень экспрессии RRM1	W	Exp (B)	р
Низкий и умеренный	Терапия митотаном	2,67	2,32	0,10
Стадия ENSAT	1,47	0,54	0,23
Высокий	Терапия митотаном	0,06	0,87	0,81
Стадия ENSAT	0,90	0,59	0,34

## ОБСУЖДЕНИЕ

АКР представляет собой редкое эндокринное злокачественное новообразование коры надпочечника с трудно предсказуемым клиническим течением и отсутствием эффективной медикаментозной терапии на поздних стадиях заболевания. Хирургическое лечение у пациентов с АКР в настоящее время является единственным возможным вариантом полного излечения опухоли [4]. Однако в ситуациях распространенного опухолевого процесса (III–IV стадия заболевания по классификации ENSAT), невозможности хирургического пособия или нерадикально выполненного оперативного лечения, рецидива заболевания, а также в случаях радикально проведенной операции (R0) и при высоком индексе пролиферативной активности в опухоли (Ki-67 более 10%), установленном после патоморфологического и ИГХ-исследования материала, безальтернативным препаратом выбора является митотан или его комбинация с цитотоксическими препаратами [3]. Основными проблемами при лечении митотаном являются его высокая токсичность и развитие целого спектра побочных явлений, что обуславливает необходимость частого и тщательного мониторинга его целевой концентрации в плазме крови. Кроме того, несмотря на то что для лечения неоперабельных случаев АКР применение митотана известно с 1959 г., влияние препарата на улучшение выживаемости все еще является дискутабельным вопросом [15]. Новый взгляд на терапию митотаном у пациентов низкого и промежуточного риска рецидива внесли результаты 3 фазы исследования ADIUVO, представленные мировому медицинскому сообществу в феврале 2022 г. на симпозиуме Американского общества клинической онкологии (ASCO) [16]. В исследование был включен 91 пациент с радикально проведенным (R0) оперативным лечением АКР, I–III стадией заболевания по классификации ENSAT и индексом пролиферативной активности Ki-67 в опухоли ≤10%, 45 из которых получали митотан в адъювантом режиме, 46 пациентов составили группу динамического наблюдения. В результате пятилетняя БРВ у пациентов низко-промежуточного риска рецидива составила около 75%, а адъювантная терапия митотаном не показала значительного положительного эффекта (8 случаев рецидива заболевания в группе пациентов с лекарственным лечением против 11 случаев рецидива заболевания в группе наблюдения).

Результаты исследования ADIUVO являются шагом к разработке системы персонализированной терапии АКР. Поиск потенциальных предикторов ответа на терапию митотаном в связи с этим является важной и актуальной задачей.

В настоящей работе мы оценили экспрессию потенциальных прогностических маркеров, таких как RRM1, SOAT1, CYP2W1, в двух группах пациентов с АКР, находящихся на динамическом наблюдении без сопутствующего лекарственного лечения и получающих терапию митотаном, и проанализировали ее связь с БРВ.

Ухудшение БРВ у пациентов, получающих терапию митотаном, при первоначальном анализе без включения уровней экспрессии RRM1, SOAT1, CYP2W1 наиболее вероятно обусловлено назначением противоопухолевой терапии более отягощенным пациентам, в том числе с более продвинутой стадией заболевания по классификации ENSAT, а не прямым негативным влиянием митотана на БРВ.

При дальнейшем анализе БРВ с учетом экспрессии потенциальных предикторов ответа на терапию митотаном выявлено, что низкая и умеренная экспрессия RRM1, SOAT1 и CYP2W1 в АКР при отсутствии противоопухолевой терапии ассоциировалась с лучшей БРВ в сравнении с пациентами, получающими лечение митотаном. В то же время при высоком уровне экспрессии факторов БРВ у пациентов контрольной и основной групп становится схожей, и статистически значимые различия БРВ пропадают. Мы предполагаем два возможных объяснения такой ситуации. Во-первых, митотан может улучшать БРВ у пациентов с высоким уровнем экспрессии обсуждаемых маркеров. То есть для пациентов с низким уровнем экспрессии митотан не оказывает достаточного эффекта, в связи с чем значение БРВ примерно соответствует общему по выборке. При этом пациенты с высоким уровнем экспрессии лучше отвечают на лечение, что позволяет «нейтрализовать» негативное влияние на выживаемость других неблагоприятных факторов у пациентов основной группы, и их БРВ становится сходной с контрольной группой. То есть назначение митотана теоретически менее благоприятным пациентам в случае высокой экспрессии обсуждаемых маркеров приближает их БРВ к теоретически более благоприятным пациентам, у которых также высокая экспрессия. Во-вторых, митотан может не оказывать влияния на БРВ. Возможно, на самом деле митотан в обеих сравниваемых группах не влияет на БРВ: пациенты контрольной группы с низкой экспрессией обсуждаемых маркеров значительно отличаются по некоему набору факторов от пациентов основной группы, поэтому их БРВ лучше. А пациенты с высокой экспрессией на самом деле имеют большое количество других факторов, повышающих риск рецидива, что приближает их к пациентам основной группы, и значения их БРВ перестают отличаться.

Далее проводилась проверка гипотезы о различной БРВ в подгруппах по митотану при разном уровне экспрессии обсуждаемых маркеров с поправкой на стадию по классификации ENSAT. Предполагается, что митотан не вызывает ухудшения БРВ в подгруппе с низкой экспрессией обсуждаемых маркеров, как в результатах однофакторного анализа. Обнаруживается, что при поправке на стадию по классификации ENSAT назначение митотана статистически значимо ухудшает БРВ из трех прогностически благоприятных групп только при CYP 1–2, в остальных случаях статистической значимости уже нет. Во всех трех группах с высокой экспрессией обсуждаемых маркеров при внесении поправки на стадию ENSAT обнаруживается, что митотан оказывает благоприятный эффект на БРВ (значение ExpB менее 1). Вместе с тем статическая значимость также не достигается, поэтому можно отметить только наличие тенденции и говорить о вероятностном характере события.

В настоящее время отсутствует единое мнение о взаимосвязи иммунореактивности потенциальных предикторов и клинического ответа на терапию митотаном при АКР. Существует мнение, что низкая экспрессия гена RRM1 при лечении митотаном вадъювантном режиме ассоциировалась с лучшей БРВ по сравнению со случаями высокой экспрессии данного маркера [10]. При анализе экспрессии гена RRM1 в клеточных линиях АКР было выявлено, что высокая активность гена RRM1 препятствует антипролиферативной активности митотана, снижая его превращение в активные метаболиты, что может лежать в основе одного из возможных механизмов лекарственной устойчивости к данному препарату [15]. Тем не менее дозозависимое увеличение транскрипции гена RRM1 под влиянием митотана, которое в исследовании ассоциировалось с отсутствием чувствительности одной из культур опухолевых клеток к воздействию терапевтического агента, требует дальнейших исследований, так как неизвестно, что именно является предиктором ответа на митотан: исходные уровни экспрессии гена RRM1 или активация его транскрипции при лечении.

CYP2W1 является цитохромом P450-монооксигеназной системы, которая участвует в биотрансформации эндогенных и экзогенных веществ, включая лекарственные препараты. Известно, что высокий уровень экспрессии данного маркера в опухоли коррелирует с более агрессивным течением заболевания [17][18]. Результаты нескольких исследований, оценивающих взаимосвязь иммунореактивности CYP2W1 и ответа на терапию митотаном в АКР, свидетельствуют, что высокая экспрессия данного маркера ассоциирована с лучшей БРВ и ОВ у пациентов, получающих терапию данным препаратом как в адъювантном, так и в паллиативном режимах [13][19].

В работе 2015 г. S. Sbiera и соавт. отметили лучшую БРВ у пациентов с АКР, находящихся на терапии митотаном, в случае высокой экспрессии SOAT1 [6]. Однако в многоцентровом ретроспективном исследовании 231 пациента с АКР, получавших митотан в адъювантом режиме, а также при распространенных формах заболевания корреляции экспрессии SOAT1 с БРВ, ОВ и опухоль-специфической выживаемостью выявлено не было [20].

В настоящее время SOAT1 и CYP2W1 рассматриваются не только как предиктивные маркеры, определяющие чувствительность АКР к воздействию митотана, но и в качестве потенциальных самостоятельных мишеней для специфической противоопухолевой терапии. Препарат Nevanimibe HCl (ATR-101), являющийся ингибитором SOAT1, в доклинических исследованиях продемонстрировал снижение стероидогенеза и индукцию апоптоза клеток надпочечника при применении в низких и более высоких дозах соответственно [21]. Однако клиническое исследование препарата у пациентов с метастатическим АКР остановлено на второй фазе вследствие невозможности достижения необходимой концентрации препарата для реализации его апоптотического действия у большинства пациентов.

Каталитическая активность CYP2W1 и его высокая экспрессия в злокачественных новообразованиях также позволяют рассматривать данный цитохром в качестве потенциальной клинически значимой мишени для специфической противоопухолевой терапии.

Таким образом, в данной работе на нашей выборке мы лишь выявили тенденции взаимосвязи экспрессии потенциальных прогностических маркеров и безрецидивной выживаемости. Изучение непосредственного влияния митотана на выживаемость требует дальнейших исследований.

## ЗАКЛЮЧЕНИЕ

АКР является редким злокачественным новообразованием эндокринной системы с труднопредсказуемым клиническим течением. Орфанный характер заболевания, а также его молекулярная и морфологическая гетерогенность в настоящее время не позволяют разработать эффективную и безопасную стратегию лечения, в связи с чем медикаментозная терапия митотаном с возможной комбинацией с цитостатическими препаратами на сегодняшний день остается практически единственным вариантом лечения пациентов.

В нашем исследовании, основываясь на мировых тенденциях и опыте зарубежных коллег, мы рассмотрели уровни экспрессии ИГХ-маркеров RRM1, CYP2W1 и SOAT1 в АКР в качестве потенциальных предикторов ответа на терапию митотаном. Результаты исследования свидетельствуют о необходимости оценки уровней иммунореактивности данных маркеров у пациентов с АКР перед началом лечения митотаном с целью прогнозирования эффективности терапии.

Прогностические особенности АКР требуют персонифицированного подхода к назначению терапии, выбор которой должен осуществляться после комплексного анализа морфологических параметров заболевания и состояния пациента. Кроме того, редкая распространенность данного заболевания диктует необходимость согласованного междисциплинарного взаимодействия эндокринологов, хирургов, патоморфологов и химиотерапевтов. Молекулярно-генетические исследования АКР, вероятно, позволят углубить понимание генеза и биологического поведения данного заболевания и усовершенствовать его диагностику и стратегии по персонализированному ведению пациентов.

## ДОПОЛНИТЕЛЬНАЯ ИНФОРМАЦИЯ

Конфликт интересов. Авторы декларируют отсутствие явных и потенциальных конфликтов интересов, связанных с публикацией настоящей статьи.

Участие авторов. Ткачук А.В. — получение, анализ данных и интерпретация результатов, написание статьи; Бельцевич Д.Г. — концепция исследования, внесение в рукопись существенной правки с целью повышения научной ценности статьи; Порубаева Э.Э. — получение, анализ данных и интерпретация результатов, написание статьи; Урусова Л.С. — концепция и дизайн исследования, получение, анализ данных и интерпретация результатов, внесение в рукопись существенной правки с целью повышения научной ценности статьи.

Все авторы одобрили финальную версию статьи перед публикацией, выразили согласие нести ответственность за все аспекты работы, подразумевающую надлежащее изучение и решение вопросов, связанных с точностью или добросовестностью любой части работы.
